# Seeing faces, when faces can‘t be seen: Wearing portrait photos has a positive effect on how patients perceive medical staff when face masks have to be worn

**DOI:** 10.1371/journal.pone.0251445

**Published:** 2021-05-19

**Authors:** Martin Wiesmann, Christiane Franz, Thorsten Sichtermann, Jan Minkenberg, Nathalie Mathern, Andrea Stockero, Elene Iordanishvili, Jessica Freiherr, Julian Hodson, Ute Habel, Omid Nikoubashman

**Affiliations:** 1 Department of Diagnostic and Interventional Neuroradiology, University Hospital RWTH Aachen, Aachen, Germany; 2 Department of Psychiatry and Psychotherapy, Friedrich Alexander University Erlangen, Erlangen, Germany; 3 Sensory Analytics, Fraunhofer Institute for Process Engineering and Packaging IVV, Freising, Germany; 4 Faculty of Business and Economics, Leuphana University of Lueneburg, Lueneburg, Germany; 5 Department of Psychiatry, Psychotherapy and Psychosomatics, University Hospital RWTH Aachen, Aachen, Germany; University of Pécs Medical School, HUNGARY

## Abstract

**Introduction:**

Since the onset of the coronavirus disease 2019 (COVID-19) pandemic, wearing surgical face masks has become mandatory for healthcare staff in many countries when interacting with patients. Recently, it has been shown that wearing face masks impairs social interaction by diminishing a person’s ability to read the emotion of their counterparts, an essential prerequisite to respond adequately in social situations. It is easily conceivable that this may have a tangible negative influence on the communication and relationship between patients and healthcare personnel. We therefore investigated whether it has an effect on how patients perceive healthcare professionals when physicians and nursing staff wear portrait photos with their smiling faces in addition to face masks.

**Methods:**

During the study period of 16 days, the medical staff of our Department wore surgical face masks at all times during any kind of interaction with patients. In a pseudorandomized order, all members of our staff additionally affixed their portrait photos to their work clothes on 8 of the 16 days. After completion of their visit, 226 patients were interviewed anonymously in a cross-sectional study design using a questionnaire in which they rated the following three items: friendliness of staff, medical quality of treatment, and how well they felt taken care of during treatment in our Department.

**Results:**

On days, on which staff wore photos, mean scores of the questionnaires were significantly higher than on non-photo days (p = 0.013; mean ± standard deviation = 92.8 ± 11.3 vs. 91.0 ± 12.6; median (range) = 97 (98) vs. 96 (76)). When analyzed separately, the increased scores were only significant for the item friendliness of staff (p = 0.009; mean ± standard deviation = 95.8 ± 6.3 vs. 92.2 ± 11.5; median (range) = 98 (39) vs. 97 (54)).

**Conclusion:**

Our study suggests that the use of portrait photos with smiling faces has a positive effect on how patients perceive healthcare staff.

## Introduction

Since the onset of the coronavirus disease 2019 (COVID-19) pandemic, most countries and health organizations such as the World Health Organization (WHO) recommend wearing face masks as one of the key measures to reduce the spread of the severe acute respiratory syndrome 2 (SARS 2) coronavirus. In many countries healthcare professionals in hospitals are presently required to wear face masks all the time when being in contact with patients.

As societies have become used to wearing masks whenever being in public or interacting with people not belonging to their own household, this trend may find long-term application beyond the current efforts in the containment of COVID-19. We may again resort to wearing masks during upcoming severe flu seasons and for potential future respiratory epidemics/pandemics. Consequently, masks may become an integral part of our daily lives for the foreseeable future. While there has been considerable research on the effectiveness of face masks to reduce viral transmission of disease, more research is needed to investigate its effects on social encounters [1, 2].

Communication between patients and healthcare personal is an essential component in clinical care. Good communication behavior during the medical interaction has been proven to be associated with a variety of satisfaction and health outcomes [3]. Communication between humans relies not only on speech but also on elements of nonverbal communication including eye contact, facial expressions, gestures, and posture. There is evidence that nonverbal information may be valued even more than verbal information in social interactions [4]. This seems also to be true for the medical setting with respect to patient satisfaction [5]. It has been shown how nonverbal communication expresses affection and forms relationship [6, 7].

Recently it has been demonstrated that wearing face masks impairs social interaction by diminishing a person’s ability to read the emotion of their counterparts [8]. We therefore hypothesized that the need to wear face masks at all times in a hospital setting may have a negative effect on the communication and in turn the binding between patients and healthcare personnel. In this experiment, we tested whether it has an effect on how patients perceive healthcare professionals when physicians and nursing staff wear portrait photos in addition to face masks.

## Materials and methods

**Fig 1:**
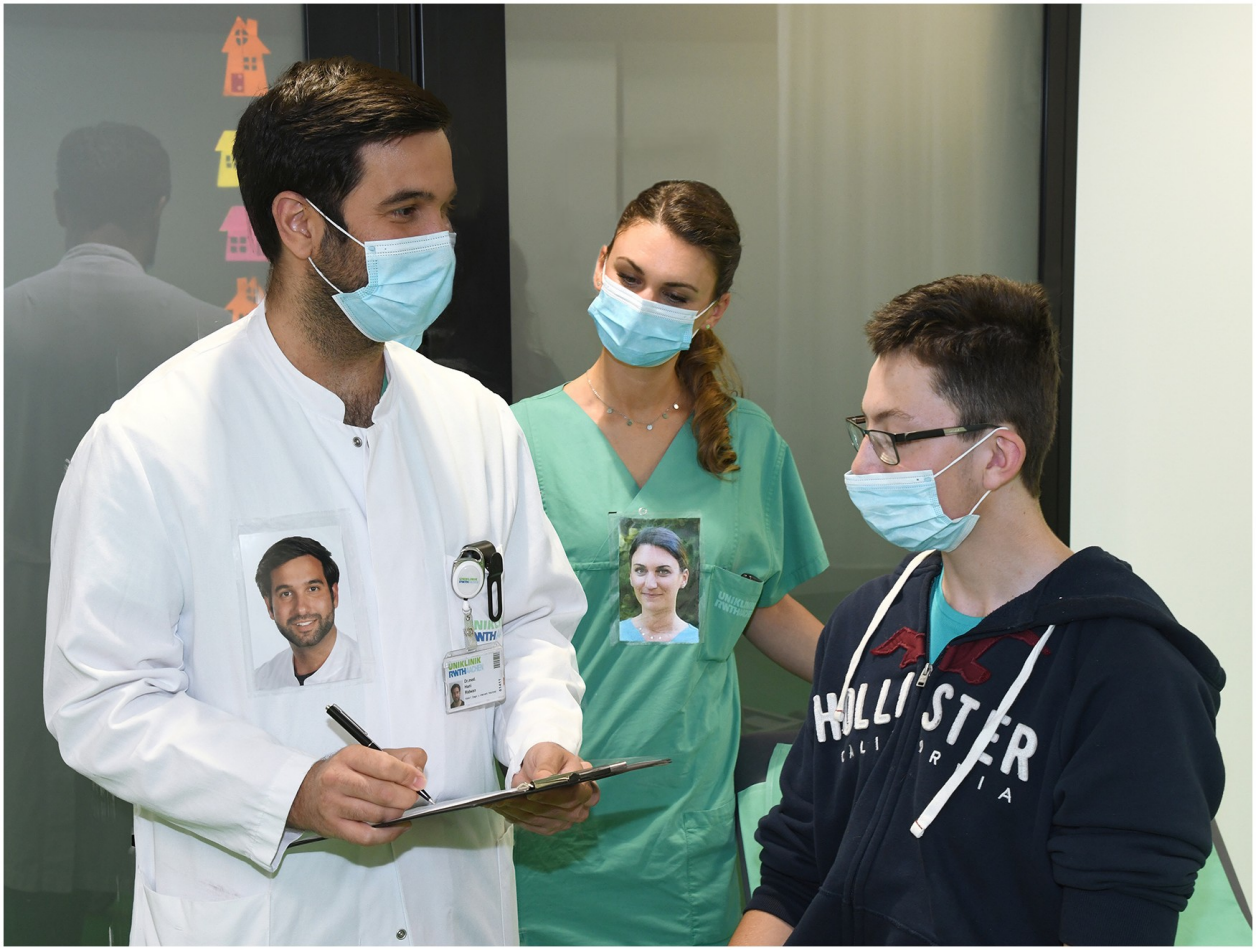
Portrait photos were placed in transparent self-adhesive sleeves and affixed to the work clothes of all staff, positioned at the chest for good visibility.

This study was part of our continuous quality control procedures and as such approved by the Ethics Committee of the University Hospital RWTH Aachen. Need for patient consent was waived since the questionnaires did not contain any personal information and were thus fully anonymous. The individuals in this manuscript have given written informed consent (as outlined in PLOS consent form) to publish these case details. The individuals pictured in Fig 1 have provided written informed consent (as outlined in PLOS consent form) to publish their image alongside the manuscript.

### Participants

The entire staff of the Department of Neuroradiology of our institution participated in the study (n = 49). This comprised 20 physicians, 19 radiographers, 4 administrative staff with patient contact, and 6 student assistants. Six research staff (PhD students, one postdoc, and one research assistant) not involved in patient care acted as interviewers.

During 16 consecutive working days from June to July 2020, all 657 patients seen by medical staff of our Department during routine working hours were considered for possible participation. We did not differentiate between inpatient and outpatient care, nor the type of treatment or consultation received. Patients were considered eligible if they were at least 18 years of age, were able to communicate with staff, and were able to understand the content of our survey. 268 patients were found to fulfil the above criteria and contacted by the research staff for participation. Of these, 230 consented to participate in the survey. 226 questionnaires were filled out correctly and could be analyzed (photo-days: n = 119; non-photo-days: n = 107).

### Portrait photographs

A professional portrait photographer took portrait photos of all staff. Subjects were instructed to smile. Photos were taken outside in natural daylight with green plants in the background. Photos were produced to postcard size (15 cm x 10 cm), placed in transparent self-adhesive sleeves and affixed to the work clothes of staff, positioned at the chest for good visibility (Fig 1).

### Questionnaires

Visual Analogue Scales (VAS) were used for the questionnaire. For VAS, straight lines are used with the endpoints defining extreme limits. For our questionnaire, the length of 10 cm for each line was used as these showed the smallest measurement error compared to 5- and 20-cm versions and seem to be most convenient for respondents [9]. In several studies, VAS have been demonstrated to be sensitive to treatment effects [9, 10].

The questionnaire addressed the three items of friendliness of medical staff, feeling well cared about, and medical quality of treatment received. In detail, the questionnaire consisted of the following three items:

1. "How friendly were our staff?"
2. "How well did you feel taken care of?"
3. "How do you rate the medical quality of our treatment?"

The questionnaires were anonymous. A small box in the lower left corner indicated whether the questionnaire was filled out on a photo day or a non-photo day.

### Survey procedures

The study ran over 16 consecutive working days. During this period medical staff wore surgical face masks at all times during patient contact. On 8 of the 16 days all staff additionally affixed their portrait photos to their work clothes in a way that they could be seen easily and in full by the patients. Days on which photos were used alternated with days on which photos were not used in a pseudorandomized order.

When patients were ready to leave the Department of Neuroradiology, i.e. after completion of their MRI study, CT study, angiographic procedure or medical consultation, patients were approached by one of the six interviewers. The interviewers were dressed in work clothes of a different color to that worn by our medical staff. They identified themselves to the patients as staff members of the hospital not being involved in patient care, and asked patients if they were willing to participate in an anonymous survey regarding the quality of treatment in the Department of Neuroradiology. If consent was given, the questionnaire was handed to the patients. Patients were instructed to mark their answers on the questionnaire unobserved by setting a cross or drawing a vertical line on the corresponding VAS, and to then put the questionnaire in a sealed box themselves.

After completion of the study the boxes were opened by the interviewers for evaluation. Visual analog scales were measured in millimeters giving three results ranging from 0 to 100 per patient. In addition, a “total” value was calculated per patient by averaging the results from all three scales.

### Statistical analysis

Statistical analyses were conducted using the statistical software Jamovi (Version 1.1.9, https://www.jamovi.org/) based on R (www.r-project.org). Normal distribution of the data was tested using the Shapiro-Wilk test. Our data were not normally distributed and were therefore submitted to nonparametric tests for independent samples (Mann-Whitney U test). P values with an alpha level of < 0.05 were considered significant. Effect sizes were calculated using the rank biserial correlation (rrb) for the Mann-Whitney U test. Within our data set there were no missing data. Therefore no specific means to handle missing data were required.

## Results

**Fig 2:**
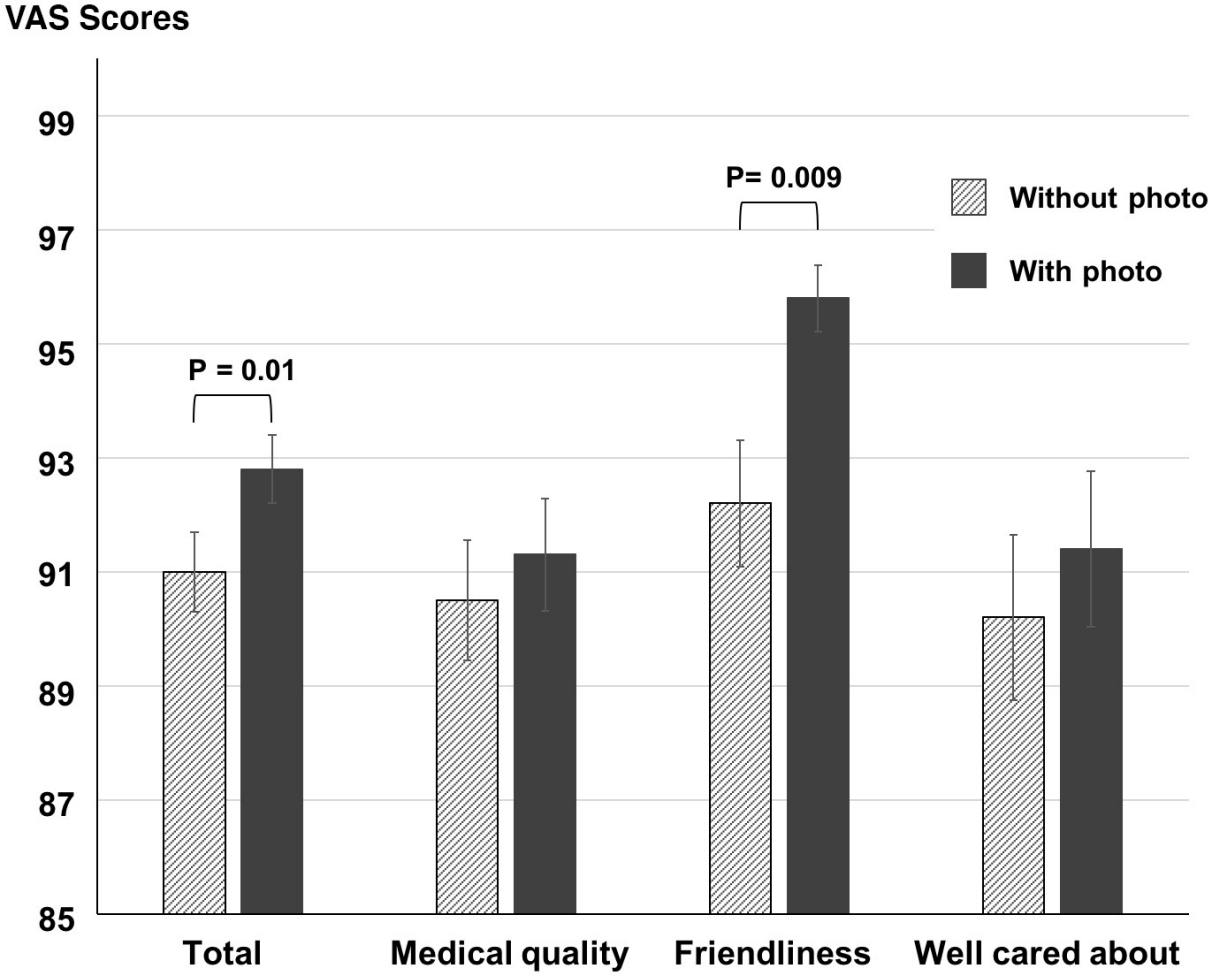
Results from a questionnaire in which 226 patients rated the following three items: Friendliness of staff, medical quality of treatment, and how well they felt taken care of during treatment in our Department using Visual Analog Scales (VAS, range 0–100, mean ± standard error of the mean). Medical staff of our Department wore surgical face masks at all times during any kind of interaction with patients. On days, on which staff additionally wore portrait photos affixed to their work clothes, mean scores of the questionnaires were significantly higher than on non-photo days (p = 0.03). When analyzed separately, the increased scores were only significant for the item friendliness of staff (p = 0.009).

On days, on which staff wore portrait photos, total scores of the questionnaires were significantly higher than on non-photo days. In detail, mean results were 92.8 ± 11.3 vs. 91.0 ± 12.6 (p = 0.013; U = 51062; Z = -2,481; effect size (Rank biserial correlation) = 0.11; means for photo days are mentioned first; median results (range) = 97 (98) vs. 96 (76)). When the three items friendliness, feeling well cared about, and medical quality of treatment were analyzed separately, scores for all items were higher on days on which staff wore photos. However, a significant difference was only found for the item friendliness (Fig 2). Mean scores were as follows: friendliness: 95.8 ± 6.3 vs. 92.2 ± 11.5 (p = 0.009; U = 5105; Z = -2,617; effect size (Rank biserial correlation) = 0.198; median results (range) = 98 (39) vs. 97 (54)); feeling taken good care of: 91.4 ± 14.9 vs. 90.2 ± 15.0 (p = 0.313; U = 5878; Z = -1,009; effect size (Rank biserial correlation) = 0.08; median results (range) = 96 (98) vs. 96 (76)); medical quality 91.3 ± 10.5 vs. 90.5 ± 11.0 (p = 0.470; U = 6015; Z = -0,723; effect size (Rank biserial correlation) = 0.06; median results (range) = 95 (45) vs. 94 (55)).

All data that support the findings of this study and detailed statistical analyses are provided in full detail in the supporting information files.

## Discussion

The COVID pandemic changed the way of interaction and communication: through wearing masks for protection it deprives us from a relevant part of facial information. This study investigated the consequences of a compensation of this lack of information by providing additional facial information via portrait photos.

To the best of our knowledge, our study is the first report evaluating if portrait photos additionally worn on clothes by healthcare staff have an effect on patients’ perception of the staff encountered and the medical treatment / consultation received. Our results indicate that the use of portrait photos with smiling faces has a positive effect on how patients perceive healthcare staff.

Communication between humans is a highly complex process involving both verbal and nonverbal components. As such, the process of speaking encompasses also nonverbal elements known as paralanguage [11]. Paralanguage includes elements such as voice quality and pitch, speech rate and loudness, the overall style of speech, as well as prosodic features such as rhythm, intonation, and stress. Moreover, humans communicate nonverbally by sending and receiving signals through e.g. eye contact, various facial expressions including particularly the mouth area of the face, gestures, posture, pupil dilation, and blink rate [12, 13].

Facial expressions are extremely important to decipher people’s emotions and intentions. In several studies it has been demonstrated that both the upper (particularly the eyes) and lower (particularly the mouth) part of the face are required for fully conveying and decoding emotional facial expressions. Precisely, the eyes and mouth represent crucial cues for detecting angry and happy expressions, respectively [14–20]. In these studies, it is a common finding that the eyes provide the most information about an emotional expression [21]. However, the mouth is also an important source of information and helps the recipient not only to distinguish between several emotions such as fear and surprise or between sadness and disgust, but also to accentuate positive emotions.

Because facial masks worn for the containment of COVID-19 fully cover the bottom half of the face, it has become considerably more difficult to interpret emotions of those wearing the masks. This holds particularly true for positive emotions such as the expression of happiness or friendliness which are largely communicated by a smile. A true or genuine smile, also known as a Duchenne smile, employs both the bottom half and the top half of the face. In order to form a Duchenne smile, one must smile with both mouth (showing one’s teeth) and eyes (through squinting) [22]. The average standardized mask typically only allows us to view the squinting half of a smile, which leaves the recipient unaware of its genuineness [23].

In addition to nonverbal information conveyed during conversation, wearing masks may limit the forming of a “first impression”. First impression formation has been shown in psychology to have an important and lasting influence on the judgment and further reception of one’s counterpart. It is formed by the initial perception of a given person, in which the overall appearance along with facial mimics play an essential role. First impressions are formed within the first seconds of contact [24]. Even though it has not been studied it appears conceivable that first impression formation is limited by not being able to see the full face of the other person.

One potential strategy, which has been proposed to overcome these limitations is the use of transparent masks. However, it has not been shown whether transparent or semitransparent masks facilitate perception of facial expressions and movements sufficiently while providing adequate protection against viral transmission. Therefore, it is conceivable that non-transparent masks will remain to be used almost exclusively.

Hence, another potential strategy could be to present a portrait photo on the clothes when face masks need to be worn. This would allow the other person to "anchor" on the face displayed on the photo during first contact or further conversation and get a better impression of the counterpart’s main facial features.

It is conceivable that changes in perception as observed in our study, and changes in first impression formation may have an effect on patients’ emotional state, or e.g. have an effect on the feeling of trust between patients and healthcare staff. Nonverbal communication is an essential part of dealing with emotions, such as the anxiety that the patient presents with. This is important since it has been shown that trust develops on the basis of verbal and nonverbal communication. Social interaction, in our context specifically the communication between patients and clinical staff, and the extent to which a feeling of trust is established, have an influence on the treatment outcome [3, 5, 25].

However, the extent to which the use of portrait photos also modulates the patients’ emotional state or has an effect on the quality of the feeling of trust between patients and healthcare staff, needs to be addressed in further studies.

Face masks act as powerful and direct hindrances to our ability to understand and empathize with one another. COVID-19 brought to the world not only a widespread disease but also a sudden and potentially lasting change in human communication. In summary, our study suggests that the use of portrait photos with smiling faces has a positive effect on how patients perceive healthcare staff, which may well have overall beneficial effects in healthcare settings. This, as well as other strategies to overcome our diminished ability to communicate when facial expressions are hidden under a mask barrier, should be followed up by further research.

### Limitations

Notwithstanding the limitations of its rather small sample size, its monocentric approach and its rather simple questionnaire design, our pilot study provides important insights for clinical practice and future research.

As outlined above this study was part of our continuous quality control procedures and as such was performed fully anonymous. Therefore we cannot provide characteristics of the study participants or potential confounders, and the generalisability of our findings may be questioned. However, since we addressed all patients seen in our Department over the full study period, our results should be representative for an average sample of hospital patients.

## Supporting information

S1 File (PDF)
