# Supplementary material for: Seeing faces, when faces can‘t be seen: Wearing portrait photos has a positive effect on how patients perceive medical staff when face masks have to be worn
Source: PLoS One. 2021 May 19;16(5):e0251445. doi: 10.1371/journal.pone.0251445 (PMC8133480; doi:10.1371/journal.pone.0251445)
Supplement: S1 File — (PDF) [file pone.0251445.s001.pdf]

SUPPLEMENTARY INFORMATION.

Supplementary Table 1. Raw data of the questionnaires for each subject.

Number of subjects	Medical quality		Friendliness		Feeling well cared about	
	No-photo	Photo	No-photo	Photo	No-photo	Photo
1	90		87		86	
2	97		95		97	
3	100		100		24	
4	100		100		100	
5	80		98		89	
6	82		98		98	
7	76		75		80	
8	95		99		99	
9	96		93		98	
10	76		83		68	
11	92		95		95	
12	83		85		79	
13	93		92		93	
14	100		98		98	
15	95		99		96	
16	98		99		98	
17	84		93		83	
18	98		73		99	
19	91		92		91	
20	100		100		100	
21	100		90		100	
22	100		100		100	
23	96		91		90	
24	66		94		64	
25	100		97		94	
26	87		86		89	
27	91		100		100	
28	91		85		91	
29	81		97		81	
30	86		90		89	
31	74		90		81	
32	95		94		100	
33	100		100		100	
34	100		100		100	
35	97		97		96	
36	75		95		95	
37	96		98		100	

**38**	100		90		95	
**39**	96		96		96	
**40**	91		100		100	
**41**	92		93		97	
**42**	93		92		90	
**43**	89		46		41	
**44**	94		100		99	
**45**	89		52		47	
**46**	100		100		90	
**47**	84		100		100	
**48**	96		67		95	
**49**	96		100		100	
**50**	96		100		91	
**51**	68		52		55	
**52**	80		85		86	
**53**	100		100		98	
**54**	85		98		98	
**55**	100		100		100	
**56**	93		95		93	
**57**	60		97		98	
**58**	100		100		100	
**59**	100		99		100	
**60**	45		47		38	
**61**	82		100		100	
**62**	100		100		100	
**63**	91		100		100	
**64**	93		94		95	
**65**	100		95		100	
**66**	96		93		71	
**67**	94		90		91	
**68**	90		95		98	
**69**	100		100		100	
**70**	89		91		92	
**71**	99		99		99	
**72**	97		97		97	
**73**	87		87		87	
**74**	90		100		98	
**75**	86		86		87	
**76**	89		93		92	
**77**	100		100		100	
**78**	68		78		56	
**79**	100		100		100	
**80**	71		84		87	
**81**	97		97		96	

**82**	98		99		98	
**83**	99		96		96	
**84**	85		99		100	
**85**	65		68		45	
**86**	96		93		93	
**87**	96		94		93	
**88**	57		57		61	
**89**	99		98		98	
**90**	88		85		85	
**91**	82		100		100	
**92**	99		97		98	
**93**	100		100		100	
**94**	79		98		64	
**95**	94		92		93	
**96**	97		97		97	
**97**	94		91		87	
**98**	97		96		95	
**99**	100		100		100	
**100**	98		99		97	
**101**	97		98		97	
**102**	67		86		94	
**103**	97		97		97	
**104**	81		90		85	
**105**	100		100		100	
**106**	91		97		91	
**107**	99		97		98	
**108**		92		97		98
**109**		72		85		93
**110**		86		89		92
**111**		100		100		100
**112**		95		94		92
**113**		80		98		84
**114**		97		100		100
**115**		79		99		90
**116**		100		100		100
**117**		78		93		82
**118**		99		99		100
**119**		100		100		100
**120**		93		93		96
**121**		99		99		97
**122**		100		100		100
**123**		100		100		100
**124**		98		95		95
**125**		98		96		99

**126**		100		100		100
**127**		70		95		67
**128**		96		96		2
**129**		100		100		100
**130**		99		99		98
**131**		92		98		98
**132**		100		100		100
**133**		88		77		87
**134**		100		100		100
**135**		97		100		71
**136**		94		91		92
**137**		100		100		100
**138**		68		61		61
**139**		98		100		89
**140**		100		100		100
**141**		100		100		100
**142**		97		96		96
**143**		100		100		100
**144**		98		98		99
**145**		97		97		97
**146**		97		96		85
**147**		92		90		92
**148**		100		100		100
**149**		93		93		96
**150**		96		100		91
**151**		100		100		100
**152**		62		80		45
**153**		90		98		98
**154**		92		93		96
**155**		97		99		93
**156**		100		100		100
**157**		87		88		88
**158**		71		86		65
**159**		87		98		88
**160**		95		98		96
**161**		100		100		100
**162**		96		98		91
**163**		93		100		100
**164**		100		100		100
**165**		91		90		83
**166**		66		100		100
**167**		95		97		95
**168**		100		100		100
**169**		83		87		97

**170**		100		100		100
**171**		88		94		94
**172**		100		100		100
**173**		80		100		89
**174**		90		92		92
**175**		91		100		99
**176**		85		97		96
**177**		98		98		98
**178**		100		99		68
**179**		95		94		94
**180**		94		91		50
**181**		87		98		57
**182**		94		92		92
**183**		96		96		94
**184**		94		94		94
**185**		69		88		86
**186**		78		81		92
**187**		92		99		99
**188**		96		95		96
**189**		100		100		100
**190**		96		96		96
**191**		77		98		100
**192**		80		82		83
**193**		87		94		92
**194**		85		89		85
**195**		87		97		94
**196**		100		100		100
**197**		66		100		99
**198**		92		93		100
**199**		90		100		100
**200**		100		100		100
**201**		90		94		91
**202**		94		89		86
**203**		95		94		60
**204**		80		99		99
**205**		88		88		85
**206**		87		100		100
**207**		90		98		97
**208**		62		94		89
**209**		57		97		71
**210**		95		96		96
**211**		82		99		99
**212**		94		99		95
**213**		55		71		28

**214**		100		100		100
**215**		100		100		100
**216**		100		100		100
**217**		85		100		92
**218**		90		93		93
**219**		100		100		100
**220**		100		100		100
**221**		100		100		91
**222**		100		100		100
**223**		97		98		98
**224**		100		100		100
**225**		99		100		100
**226**		93		93		96

Supplementary Table 2. Descriptive U-statistics.

	Photo*	Medical quality	Friendliness	Well cared about	Total
N	0	107	107	107	321
	1	119	119	119	357
Missing	0	0	0	0	0
	1	0	0	0	0
Mean	0	90.49	92.24	90.24	90.99
	1	91.29	95.76	91.42	92.82
Median	0	94	97	96	96
	1	95	98	96	97
Standard error mean	0	1.059	1.109	1.452	0.704
	1	0.959	0.575	1.366	0.597
Standard deviation	0	10.96	11.47	15.02	12.60
	1	10.46	6.269	14.91	11.28
Variance	0	120.1	131.5	225.7	158.9
	1	109.4	39.30	222.2	127.2
Range	0	55	54	76	76
	1	45	39	98	98
Minimum	0	45	46	24	24
	1	55	61	2	2
Maximum	0	100	100	100	100
	1	100	100	100	100
Shapiro-Wilk p	0	< .001	< .001	< .001	< .001
	1	< .001	< .001	< .001	< .001

Photo*: 0 = without photo; 1 = with photo.

References:

The jamovi project (2020). *jamovi*. (Version 1.2) [Computer Software]. Retrieved from https://www.jamovi.org.

R Core Team (2019). *R: A Language and environment for statistical computing*. (Version 3.6) [Computer software]. Retrieved from https://cran.r-project.org/.

Kerby, D. S. (2014). The simple difference formula: An approach to teaching nonparametric correlation. *Comprehensive Psychology, 3*, 2165–2228.

Supplementary Table 3. Non-parametric independent sample T-test.

		Statistic	p		Effect Size
Medical quality	Mann-Whitney U	6015	0.470	Rank biserial correlation	0.05529
Friendliness	Mann-Whitney U	5105	0.009	Rank biserial correlation	0.19823
Well cared about	Mann-Whitney U	5878	0.313	Rank biserial correlation	0.07673
Total	Mann-Whitney U	51062	0.013	Rank biserial correlation	0.1089

References:

The jamovi project (2020). *jamovi*. (Version 1.2) [Computer Software]. Retrieved from https://www.jamovi.org.

R Core Team (2019). *R: A Language and environment for statistical computing*. (Version 3.6) [Computer software]. Retrieved from https://cran.r-project.org/.

Kerby, D. S. (2014). The simple difference formula: An approach to teaching nonparametric correlation. *Comprehensive Psychology, 3*, 2165–2228.
